# Predicting CRISPR/Cas9 Repair Outcomes by Attention-Based Deep Learning Framework

**DOI:** 10.3390/cells11111847

**Published:** 2022-06-05

**Authors:** Xiuqin Liu, Shuya Wang, Dongmei Ai

**Affiliations:** School of Mathematics and Physics, University of Science and Technology Beijing, Beijing 100083, China; b0001428@ustb.edu.cn (X.L.); s20190757@xs.ustb.edu.cn (S.W.)

**Keywords:** DNA repair, deep learning, positional encoding, attention mechanism

## Abstract

As a simple and programmable nuclease-based genome editing tool, the CRISPR/Cas9 system has been widely used in target-gene repair and gene-expression regulation. The DNA mutation generated by CRISPR/Cas9-mediated double-strand breaks determines its biological and phenotypic effects. Experiments have demonstrated that CRISPR/Cas9-generated cellular-repair outcomes depend on local sequence features. Therefore, the repair outcomes after DNA break can be predicted by sequences near the cleavage sites. However, existing prediction methods rely on manually constructed features or insufficiently detailed prediction labels. They cannot satisfy clinical-level-prediction accuracy, which limit the performance of these models to existing knowledge about CRISPR/Cas9 editing. We predict 557 repair labels of DNA, covering the vast majority of Cas9-generated mutational outcomes, and build a deep learning model called Apindel, to predict CRISPR/Cas9 editing outcomes. Apindel, automatically, trains the sequence features of DNA with the GloVe model, introduces location information through Positional Encoding (PE), and embeds the trained-word vector matrixes into a deep learning model, containing BiLSTM and the Attention mechanism. Apindel has better performance and more detailed prediction categories than the most advanced DNA-mutation-predicting models. It, also, reveals that nucleotides at different positions relative to the cleavage sites have different influences on CRISPR/Cas9 editing outcomes.

## 1. Introduction

The CRISPR/Cas9 system is derived from the type II CRISPR/Cas system, which provides adaptive immunity to viruses and plasmids for bacteria [1,2,3]. The full name of CRISPR is Clustered Regularly Interspaced Short Palindromic Repeats [4], and these repetitive sequences are separated by distinct non-repetitive sequences called spacers. When an exogenous virus invades the host, the viral DNA is processed by Cas nuclease, into small DNA fragments, which are then integrated into the CRISPR of the host genome as spacers. The spacers are used as the transcriptional templates to produce crRNA, and the mature crRNA with tracrRNA form a special RNA structure (gRNA), through base complementary pairing, which guides the cas9 protein to recognize the specific site (PAM motif) [5,6] of the target DNA, causes double-strand breaks, and enables targeted editing of the genome [7]. Since cells cannot survive long with their DNA cleavage, their alarms go off, as soon as the DNA is broken. The steps to repair the break begin quickly.

DNA double-strand breaks are, primarily, repaired by one of two pathways: non-homologous end-joining (NHEJ) and homology-directed repair (HDR) [8,9]. In addition to this, there is evidence for microhomology-mediated end-joining (MMEJ). HDR [10] is complex and precise, can only occur in the *G2* and *S* phase of the cells, and repairs double-strand breaks (DSBs), using homologous template sequences, but it is inefficient. In contrast, NHEJ [11] is rapid and imprecise, directly rejoining broken ends, usually in the form of short insertions or deletions (indels), which occur throughout the cell cycle. Unlike classical NHEJ, MMEJ [12] relies on regions of microhomology, to repair breaks. MMEJ repair of broken DNA, often, results in the deletion of micro-homologous fragments at the junction or, even, leads to gene rearrangements, so it is an error-prone repair method. Recent studies have shown that the repair outcomes of NHEJ and MMEJ are determined by the sequence characteristics of the target DNA [13].

Owing to the fact that mutational outcomes vary with cell lines and Cas9 modifications [14], accurate prediction of template-free CRISPR/Cas9 editing outcomes is, undoubtedly, a challenging bioinformatics problem. Thanks to rapid advances in machine learning, researchers better conducted CRISPR/Cas9 studies. Various machine-learning-based prediction tools have been developed and put into use (Table 1).

inDelphi [15] introduced different sequence features, such as microhomology length, built three interconnected modules based on the deep neural network or k-Nearest Neighbor algorithm, and, then, predicted MH deletions, MH-less deletions, and 1 bp insertions, respectively. SPROUT [16] took the 20 nucleotides of the spacer sequences plus the PAM, as inputs, and built a Gradient Boosting Decision Tree model. At each target site, the model predicted nine statistics, such as the average deletion length. CROTON [17] is a deep learning framework based on the Convolutional Neural Network (CNN) and Neural Architecture Search (NAS), which predicted 1 bp insertion and deletion probability as well as deletion and frameshift frequency, from raw one-hot encoded DNA sequences. Several models mentioned above predicted only a few fixed repair labels during model building, which cannot cover all mutational outcomes and have insufficient prediction accuracy. FORECasT [14] generated candidate mutations for each gRNA and derived features for them based on local sequence characteristics, training a multi-class Logistic Regression model designed to predict repair outcomes. Lindel [18] defined binary features to characterize the target sequences and used one-hot encoding to convert the sequences to matrixes as model inputs, so three components were trained independently using different Logistic Regression models aiming to predict the ratio of insertions to deletions, the distribution of deletion events, and the distribution of insertion events. Although the above two models can predict the vast majority of mutational outcomes, the inherent mechanism of CRISPR gene editing technology is, still, unclear, and both the artificial construction of a set of binary features describing the sequences or mutational information alone and the conversion of the sequences into sparse matrixes using unique one-hot encoding as model inputs may, negatively, affect the prediction performance.

Here, we developed a model named Apindel (a deep learning framework based on the Attention mechanism and Positional Encoding, for predicting CRISPR/Cas9 repair outcomes) to address the problem of predicting DNA mutations, using contextual sequences surrounding the cleavage sites. First, through introducing the GloVe embedding model, the cooccurrence matrixes were constructed to extract the global statistical information of the input sequences; second, the target sequences were converted into dense matrixes, by combining the Positional Encoding; finally, the model was combined with the deep neural network model, adding an attention layer, which can reveal that the bases at different sequence loci have varying degrees of effect on predicting DNA repair outcomes. Apindel can, accurately, predict the mutational outcomes for a given target sequence. We demonstrated that Apindel, which was trained on the FORECasT dataset, outperformed existing approaches on the most prediction tasks. Therefore, it is expected to be a potential tool to aid research on CRISPR systems.

## 2. Materials and Methods

### 2.1. Data Sources

To date, there is no public website to integrate large-scale mutational profiles [19]. Most studies, still, use various detection methods to measure editing produced by gRNAs in synthetic constructs and to study the effect of flanking DNA target sequences on repair outcomes. Since Lindel [18] was the only published paper to date that provided data corresponding to 557 repair labels, we used the data that has been published in the Lindel article as our training data. Lindel obtained a large number of repair outcomes, by high-throughput sequencing, which was analyzed and aggregated to result in a dataset of~1 million UMIs, representing 4790 target sequences. The 4790 target sequences in the modeling dataset were split into subsets of 3900 (for training), 450 (for validation), and 440 (for testing), to ensure that the training, testing, and validation sets were consistent with the Lindel model. We, also, selected 4298 targets in the ForeCasT [14] test set, as an independent dataset for the test of generalization ability. 

We selected the dataset from the FORECasT-improved scaffold [14] as the baseline data (because FORECasT provided one of the largest datasets of CRISPR/Cas9 editing outcomes, relative to inDelphi [15], SPROUT [16], and Lindel [18]), with a total of 35,129 target sequences. The data were randomly divided into training, validation, and test data, in a ratio of 8:1:1. The training set was used for the construction of Apindel, the validation set was used to monitor the model training convergence and early stopping, while the test set was used to evaluate the prediction performance of the training model.

We, also, selected the test set of SPROUT [16], as an independent, unseen dataset, for the evaluation of the generalization ability of the model, with a total of 1603 target sequences. To obtain the DNA sequences for SPROUT, the retrieved genomic coordinates were mapped to the human genome construct 38 (hg38).

### 2.2. Data Preprocessing

For the above datasets, we truncated 60 bp genomic sequences as the model inputs. Specifically, for each DNA target sequence in the datasets, we aligned the PAM site at 33nt, so that the cut site was located at the center (30nt) of all input sequences. For sequences less than 60 bp, we considered adding “ATG” before the reverse sequences and “ATGC” after the forward sequences (see Supplementary Note, for more details).

In order to compile repair outcomes comprehensively and model efficiently, we considered a total of 557 repair labels for prediction. Specifically, since large mutational events rarely occur, we restricted the deletion length to <30 bp and overlapped with the window (from 3 nucleotides upstream to 2 nucleotides downstream of the cut site), for a total of 536 deletion labels, considering the sites where the deletion events occurred as well as the length. The insertion labels contain 4 possible single nucleotide insertions and 16 possible dinucleotide insertions and ≥3 bp insertions, for a total of 21 insertion labels [18]. For example, “−5 + 6” means a deletion occurs at 5 nucleotides upstream of the cleavage site, and the deletion length is 6 bp.

**Figure 1:**
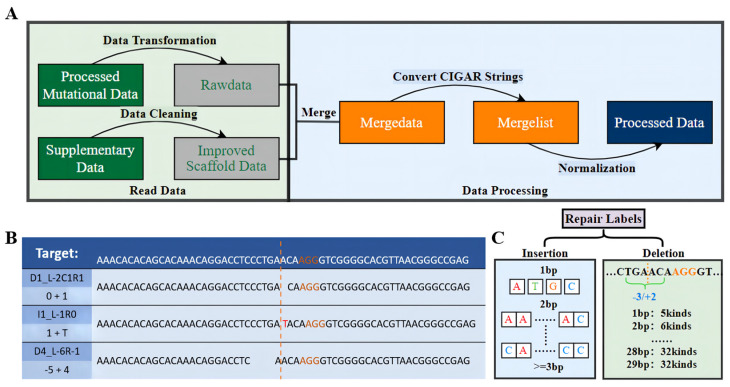
Overview of data preprocessing. (**A**) Data preprocessing process from left to right. (**B**) Example of conversion of CIGAR strings. The yellow dashed line indicates the cut site, the yellow font represents the PAM, the top is the DNA sequence of the editing target, and the bottom is the repair labels corresponding to the three mutational outcomes. (**C**) The 557 repair labels, including insertions and deletions.

For preprocessing the FORECasT data (Figure 1A), first, we converted the content and format of the mutational outcomes obtained from large-scale sequencing, to obtain the initial dataset. Second, data cleaning was performed on the SupplementaryData1 mentioned in the FORECasT article, and the ID, GuideSequence, and TargetSequence corresponding to the improved scaffold data were selected, to obtain the improved scaffold dataset. Then, the two aforementioned datasets were merged according to the ID, converting the reverse sequences and their mutational outcomes into forward sequences, to obtain the merged dataset. Next, the conversion of the CIGAR string was performed (Figure 1B). For example, “D1_L-2C1R1” and “0 + 1” represent the same repair outcome, while converting “D1_L-2C1R1” to “0 + 1” and “I1_L-1R0” and “1 + T” indicate the same repair result, and “D4_L-6R-1” and “−5 + 4” represent the same repair result. Finally, we needed to collate the mutational outcomes obtained from large-scale sequencing into 557 repair labels (Figure 1C), adjusted the data format, selected the 60 bp sequence as the model inputs, aligned the PAM site at 33nt, and performed the normalization operation, so that the sum of the frequencies of mutational labels for each DNA sequence is one. The data processing for the SPROUT test set was similar to the operation in the CROTON paper [17], and was not repeated here. For each DNA sequence, the following edit-outcomes statistics were calculated: 1 bp insertion frequency, 1 bp deletion frequency, deletion frequency, removed outliers, and missing values. For the Lindel [18] dataset, we directly selected the data from published papers.

### 2.3. Data Encoding

#### 2.3.1. Global Vectors for Word Representation

GloVe [20] is a global log bilinear regression model, an unsupervised learning algorithm for obtaining vector representations of words. Considering the global features of the corpus and that the words are represented by vectorization, the vectors contain as much semantic and syntactic information as possible between them. First, the model trained on aggregated global word–word cooccurrence statistics from the corpus, and, then, it learned word vectors based on the cooccurrence matrix and the GloVe model, as input vectors to the neural network. The model-result representation shows an interesting linear substructure of the word-vector space. GloVe model is based on global prior statistical information from matrix decomposition, coupled with local information from text-box restrictions, which both speed up the training of the model and limit the relative weights of words.

In order to implement the GloVe embedding model, for a sequence of given length $L_{0}$, the whole sequence was scanned with a sliding window of window length Kmer=K and sliding step size Kstep=s, before it was split into multiple *k-mer* based on the information intercepted by each window, to obtain a *k-mer* sequence of length $L=\left[\left(L_{0}-K\right)/s\right]+1$. Given each *k-mer*, an index corresponds to the set of positive integers $\mathbb{Z}=\left[1,2,\cdots ,4^{K}\right]$. For all *k-mer* sequences obtained, a transformation dictionary of *k-mer* embedding vectors was obtained, using the GloVe word-vector-generation model, which, with the help of a single *k-mer*, could be transformed into a vector, with the whole *k-mer* sequence transformed into a matrix for subsequent calculations.

#### 2.3.2. Positional Encoding

Experiments showed that nucleotides at different positions in the sequence have a preference for repair outcomes [16,21]. Positional Encoding vectors are added to the embedding, to convey position information throughout the model. Positional Encoding [22] is a method that uses the position information of the word to represent each word in a sequence secondarily. While the original input to the model is the word vector without word order information, Positional Encoding requires the combination of word order information and word vectors to form a new representation input to the model, so that the model has the ability to learn word-order information.

In this work, we used the sine and cosine functions [23]. Given an input sequence of length *n*, *t* denoted the position of the word in the sequence, $\vec{p_{t}}\in {\mathbb{R}}^{d}$ represented the vector corresponding to position *t*, and *d* was the dimension of the vector. $f:\mathbb{N}\to {\mathbb{R}}^{d}$ was a function that generated position vector $\vec{p_{t}}$, defined, as follows:

(1) $${\vec{p_{t}}}^{\left(i\right)}=f{\left(t\right)}^{\left(i\right)}:f\left(x\right)=\{\begin{array}{c} \sin\left(\omega_{k}\cdot t\right),\;\;\;\;\;\;\;\;\;\;if\;i=2k\\ \cos\left(\omega_{k}\cdot t\right),\;\;\;\;\;\;\;\;\;\;if\;i=2k+1 \end{array}$$

where frequency $\omega_{k}$ was defined, as follows:

(2) $$\omega_{k}=\frac{1}{10000^{2k/d}}$$

It followed from the definition of the function that the frequency decreased along the vector dimension. In order for each word vector to have its positional information, Positional Encoding was added to the model inputs. Specifically, for each word $\omega_{t}$ in the sentences, to calculate its corresponding word embedding $\psi \left(\omega_{t}\right)$, the model input was, then, obtained, as follows:

(3) $$\psi^{\prime }\left(\omega_{t}\right)=\psi \left(\omega_{t}\right)+\vec{p_{t}}$$

To ensure that this summation operation was correct, let the dimension of the Positional Encoding be equal to the dimension of the word encoding (WE), i.e., $d_{WE}=d_{PE}$. Instead of a single value, this encoding was a *d*-dimensional vector, containing information about a particular position in the sentence; instead of integrating into the model, this encoding used this vector to represent information about the position of each word in the sentence. In other words, the model input was augmented, by injecting information about the order of the words.

In the GloVe model, the sequence of a given length $L_{0}$ had been transformed to *k-mer* sequence of length $L=\left[\left(L_{0}-K\right)/s\right]+1$. To implement Positional Encoding, giving each *k-mer* an index corresponding to the set of positive integers ℤ=[1,2,⋯,L], we considered a vector representation of L positions, ensured that the dimension of the location Positional Encoding was equal to the dimension of the word encoding, and obtained a transformed dictionary of location vectors, with which the information of the different loci can be represented for subsequent computations.

### 2.4. Deep Learning Algorithm

#### 2.4.1. Bidirectional Long Short-Term Memory

Long Short-Term Memory (LSTM) is a type of Recurrent Neural Network (RNN). Since LSTM can learn which information to remember and forget through training, the LSTM model can capture longer-distance dependencies well, but the LSTM model, also, has a certain limitation: it cannot capture information from back to front. We consider using the BiLSTM model, Bi-directional Long Short-Term Memory (BiLSTM), which is composed of forward LSTM and backward LSTM. BiLSTM allows for better capture of bidirectional semantic dependencies.

#### 2.4.2. Attention Mechanism

The Attention mechanism [23] is a technique that enables models to focus on important information and learn it sufficiently, which can be applied to any sequence model. In contrast to conventional encoding methods, such as Convolutional Neural Networks (CNN) and RNN, which employ various pooling or, directly, take the hidden state of the last *t*-moment as the vector output of the sentence and do not pay special attention to the location information, the Attention mechanism provides a set of weights that reflect the distinct degree of attention given to different morphemes in a sentence sequence, through calculation.

To capture the important contextual sequence features involved in the determinants of DNA repair outcomes, we incorporated an attention layer in our model, given the feature matrix $H=\left(h_{1},\cdots ,h_{L}\right)$, we computed the attention score $a_{i}$ of the *i*th position in the attention vector $a={\left(a_{1},\cdots ,a_{N}\right)}^{T}$ by

(4) $$\;a_{i}=\frac{exp\left(w_{2}^{T}f\left(h_{i}\right)\right)}{\sum_{i=1}^{L}exp\left(w_{2}^{T}f\left(h_{i}\right)\right)},$$

(5) $$f\left(h_{i}\right)=tanh\left(W_{1}h_{i}\right)$$

where $W_{1}\in {\mathbb{R}}^{T\times C}$ stands for a weight matrix (*T* is a hyperparameter that needs to be determined, and *C* is the dimension of the feature matrix) and $w_{2}$ represents a weight vector. Next, we multiplied the attention vector $a^{T}=\left(a_{1},\cdots ,a_{N}\right)$ with the original feature matrix *H* and, then, fed the result into a multilayer perception (MLP) network, followed by a SoftMax activation function.

### 2.5. Model Training

We trained the GloVe model, by calling the Python extension package mittens, usually setting the truncation values of the co-occurrence matrix (XMAX) to the average of the elements in the co-occurrence matrix and the set parameters ensure that (60−KMER)/Kstep was an integer. Call torch, to build Positional Encoding, vector dimensions, and Dropout parameter *p* were the hyperparameters that we needed to set. Building a deep learning framework with Keras (Table S1), in order to prevent the model from encountering the overfitting problem, we added L1 regularization, Batch Normalization, and Dropout layers to the model. Since each target sequence can produce many possible repair outcomes, we trained our model with soft labels (i.e., the probability that each sequence corresponds to each category), instead of hard labels (i.e., each sequence can only correspond to one repair category). The model was trained using the Adam optimizer, with the learning rate set to 0.0001, the loss function set to categorical_crossentropy, and the evaluation metric set to mse. In order to obtain a model with good performance, many hyperparameters needed to be set during training, such as epoch. We chose EarlyStopping and ReduceLROnPlateau to solve the problem that the number of epochs needed to be set manually. We selected the best model based on performance on the validation set according to the coefficient of determination using grid search over hyperparameters. This search included coefficients for L1 regularization ranging from $10^{-4}$ to $10^{-7}$, parameters for the Dropout layer (*p* = 0.5,0.6), batch size (64, 128 or 256), scaling learning rate (factor) for ReduceLROnPlateau, patience (ReduceLROnPlateau), and patience (EarlyStopping).

We implemented the proposed methods in Python 3.8.8 and Keras library 2.4.3. The training and testing processes were performed on a desktop computer with Intel^®^ Xeon (R) Gold 5118 CPU @ 2.30 GHz × 45(Intel, Santa Clara, CA, USA), Ubuntu 16.04 LTS (GNU/Linux 4.15.0-142-generic x86_64) and 125 GB RAM. Two NVIDIA TITAN Xp per GPU have been used to accelerate the training and testing process.

### 2.6. Performance Testing of Machine Learning Models

In order to test the model prediction performance, we selected Lindel as the control models. We selected the same 440 sequences as Lindel, as a test set, to examine the prediction performance of both Apindel trained on the Lindel dataset and Lindel on this dataset. To test the generalization ability of Apindel trained on the Lindel dataset, we chose the independent FORECasT dataset as the test set.

In addition, we selected CROTON as the control models. First, we chose the same 3512 sequences as CROTON, as the test set, and we applied Apindel trained on the FORECasT dataset to this test set. Second, we selected six prediction tasks for model comparison, namely 1 bp insertion frequency, 1 bp deletion frequency, deletion frequency, 1 bp frameshift frequency, 2 bp frameshift frequency, and overall frameshift frequency. Since Apindel could predict the repair labels covering almost all possible unique mutational outcomes, when checking the accuracy of the model, we needed to convert the prediction outcomes of Apindel into the set-prediction tasks. For the CROTON model, we directly selected the results from the published papers [17].

To test the generalization ability of Apindel trained on the FORECasT dataset, we selected the reserved SPROUT dataset and Lindel dataset, as the test set, and compared with SPROUT, inDelphi, CROTON, FORECasT, and Lindel. The comparison on the SPROUT dataset was similar to the accuracy validation described above, and we collated the prediction outcomes of Apindel, FORECasT, and the Lindel model into selected prediction tasks. For the CROTON model, we, directly, selected the results from published papers [17]. For inDelphi model, metrics are reported for the best-performing model on HEK293 cell lines [15]. For the SPROUT model, we compared the performance of Apindel with published metrics from SPROUT (i.e., Kendall tau for 1 bp insertion and deletion probabilities and Pearson’s correlation for deletion frequency). For the Lindel test set, we applied Apindel and CROTON to this dataset, for model comparison.

We selected Mean Square Error (mse), Area Under Curve (AUC), the Pearson correlation coefficient, and Kendall tau to evaluate the performance of Apindel. AUC was calculated by whether the model made accurate predictions above or below the median value, in each task dataset (see Supplementary Note, for more details).

## 3. Results

### 3.1. Apindel Architecture

**Figure 2:**
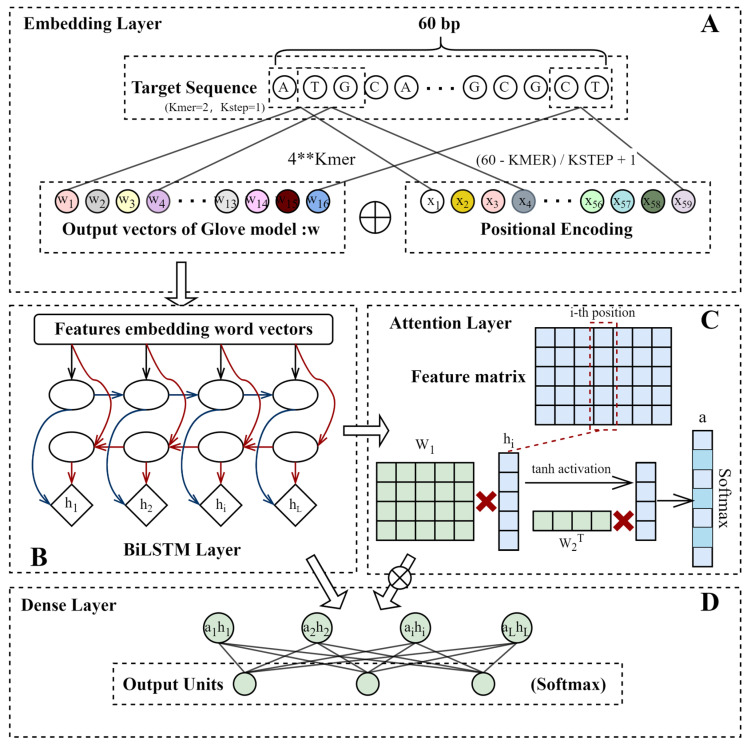
Apindel architecture. (**A**) Embedding layer, mainly consisting of GloVe model and Positional Encoding. (**B**) BiLSTM layer, used to capture the contextual information in the input message. (**C**) Attention layer. The feature matrix output from the previous layer calculated the attention vector, which stored the importance scores of different loci of the sequences on the final prediction outcomes. (**D**) Dense layer. The attention vector was combined with the BiLSTM layer output, and the final, fully connected layer was used to obtain the final results.

Based on the previous research findings, that “repair outcomes can be predicted by the local context of the sequences” [19,21,24], we constructed the Apindel model (Figure 2), which could accurately predict the outcomes and repair probabilities, based on the sequences near the cleavage sites. Considering the efficiency and accuracy of the model, we truncated 60 bp genomic sequences as model inputs, and we described the mutational outcomes as model outputs, with a total of 557 labels considered.

The first layer of the model was the embedding layer. The *k-mer* corpus was obtained, by setting the sliding window size *Kmer* to 2 and the sliding step length *Kstep* to 1, so the size of the *k-mer* vocabulary was, thus, $N=4^{2}=16$, and the indexes corresponded to the set of positive integers ℤ=[1,2,⋯16]. We transformed the input target sequences into the *k-mer* sequences of length L=[(60−2)/1]+1=59, resulting in a location information vocabulary of size M=59, with indexes corresponding to the set of positive integers ℤ=[1,2,⋯59]. The *k-mer* were combined with the position information, to obtain a vocabulary of size vocabsize=N∗M=944, and the indexes corresponded to the set of positive integers ℤ=[1,2,⋯944]. The vector-conversion dictionary was trained through the GloVe model and Positional Encoding, respectively, the corresponding GloVe word vector and the positional vector were added according to the index, and, subsequently, the individual *k-mer* was transformed into a vector, so the whole *k-mer* sequence was converted into the matrix, as the input to the deep learning model. We called the Python extension package mittens to train the GloVe model, based on our own code implementation of the GloVe cooccurrence matrix. Regarding the hyperparameters of the GloVe word-vector-generation model, the dimension of the embedding vector was set to 150, the size of the window diameter for calculating the cooccurrence matrix was set to 5, the truncation value in the auxiliary function was set to 10,000, and the maximum number of iterations was set to 20,000. We called the torch to build the PE model, and the dimension of the embedding vector was set, as the same as the GloVe model: dropout parameter (*p* = 0).

The second layer was the BiLSTM network, which was, mainly, used to extract the contextual features of the input information. The number of neurons (*units*) was set to 50, the activation function was ReLU, and L1 regularization $\left(\lambda =10^{-4}\right)$ was added to this layer. The output of the layer was used for the computation of the next layer and the final result.

The third layer was the Attention layer, and the computational process of this layer was divided into three steps. First, we calculated the weight matrix, multiplied it with the feature matrix output from the previous layer, and chose the activation function as tanh. Then, we calculated the weight vector, multiplied it with the matrix output by the previous step, selected the activation function as SoftMax, and obtained the attention vector *a*. Finally, the attention vector was multiplied by the feature-matrix input from this layer, and, then, the result was fed into a multilayer perception (MLP) network, followed by the SoftMax activation function. We added L1 regularization $\left(\lambda =10^{-4}\right)$ to this layer. The prediction results of the model were obtained.

During the training process, we used the Adam optimizer [25] with a learning rate of 0.0001, to optimize the loss function. In order to avoid the occurrence of overfitting, we set the batch size to 64 and used the categorical cross-entropy loss. The factor of the callback function ReduceLROnPlateau was set to 0.2, training proceeded for a maximum of 100 epochs, with a “patience” of 1 and a “patience” of 3, meaning that training shrunk the learning rate after one epoch and stopped after three epochs, with no improvement in validation-set performance. The detailed description of the model structure is in the Supplementary Material.

### 3.2. Model Selection

We evaluated the predictive performance of Apindel against other pre-selected models (Table 2). The structure of the benchmark model Apindel was described in the “Apindel architecture” section. Apindel_NoPE was obtained, by removing the Positional Encoding part from the data encoding of the Apindel model; Apindel_Noattention was the Apindel model, encoded without the Attention mechanism. First, all models were trained on the Lindel dataset and were evaluated on the test set using Mean Square Error (MSE). Second, all models were trained on the FORECasT dataset and were evaluated on the test set using Area Under Curve (AUC) and Pearson correlation coefficients; the relevant data descriptions are described in the “Data Sources” section.

Compared with the Apindel_NoPE model and the Apindel_Noattention model (Table 3), we can clearly see that Apindel achieved better prediction performance, on the Lindel test set.

**Figure 3:**
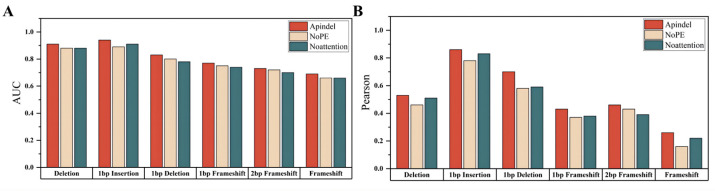
Performance evaluation of pre-selected models on the FORECasT dataset. (**A**,**B**) Apindel performed well under both evaluation metrics combined. The horizontal coordinates were the pre-selected models and the prediction tasks, and the vertical coordinates were the results under AUC and Pearson, respectively.

Compared with the benchmark model, we can see that the Apindel model predicted the six prediction tasks used for model comparison, with much better accuracy than the Apindel_NoPE model and the Apindel_Noattention model (Figure 3), The AUC values for predicting Deletion frequency and 1 bp Insertion frequency were both greater than 90% (Table S2). In summary, the position information was very important to our model, and assigning different weights to different sequence loci, through the Attention mechanism, was helpful to improve the prediction of the model.

### 3.3. Model Comparison

Recently, five machine-learning models have been developed to predict the outcome of DNA sequence repair, namely SPROUT [16], inDelphi [15], CROTON [17], FORECasT [14], and Lindel [18]. SPROUT was a Gradient Boosting Decision Tree model that predicted nine repair types, including average deletion length, and provided an independent dataset of CRISPR/Cas9 editing results. CROTON utilized NAS, to automatically create a multi-task deep Convolutional Neural Network framework, for prediction of CRISPR/Cas9 editing outcomes, which can predict six repair types, including deletion frequencies. inDelphi built three interconnected modules, based on neural networks and k-Nearest Neighbors, aiming to predict microhomology (MH) deletions, MH-less deletions, and 1 bp insertions. FORECasT built a multi-class Logistic Regression model, capable of predicting all possible unique repair outcomes, and it provided one of the largest datasets of CRISPR/Cas9 editing results, relative to inDelphi, SPROUT, and Lindel, for the establishment of Apindel. Lindel was a Logistic Regression model; unlike the above models, it provided an exact category criterion for predicting repair outcomes, including insertion and deletion sites, insertion nucleotide types, and deletion length.

For a more comprehensive comparison with the five models mentioned above, we applied the Apindel model, trained on the Lindel dataset, to the Lindel test set and the FORECasT test set, and evaluated the predictive performance of the model using the mean squared error (mse). The Apindel model trained on the FORECasT dataset was applied to the randomly selected FORECasT test set, the SPROUT test set, and the Lindel test set, and the predictive performance of the models was evaluated using the Kendall’s tau rank correlation coefficient, Pearson correlation coefficient, or AUC between predicted and observed values (Table S3).

#### 3.3.1. Evaluation of Predictive Performance on 557 Predictive Labels

First, since Lindel was the only model in the published papers, so far, that predicted 557 repair outcomes, we compared the Apindel model trained on the Lindel data with Lindel. We trained on the Lindel dataset and predicted the frequency of 557 classes of indels. Comparing the results of our model and Lindel on the test set, our model worked better (MSE = 0.000164 and 0.000172 for our model and Lindel, respectively). The above results illustrated that our model had better performance, in predicting 557 repair outcomes. To validate the generalization ability of Apindel, we analyzed the prediction performance of Apindel on the independent FORECasT dataset. Predicting 557 classes of indels on the FORECasT dataset did not work as well as on the Lindel test set (MSE = 0.000197). Based on the previous research findings, that “the overall distribution of repair outcome types was not the same across the different cell lines” [14], we speculated that this problem may be caused by differences in repair outcomes between different cell lines.

#### 3.3.2. Evaluation of Predictive Performance on Six Classes of Prediction Tasks

**Figure 4:**
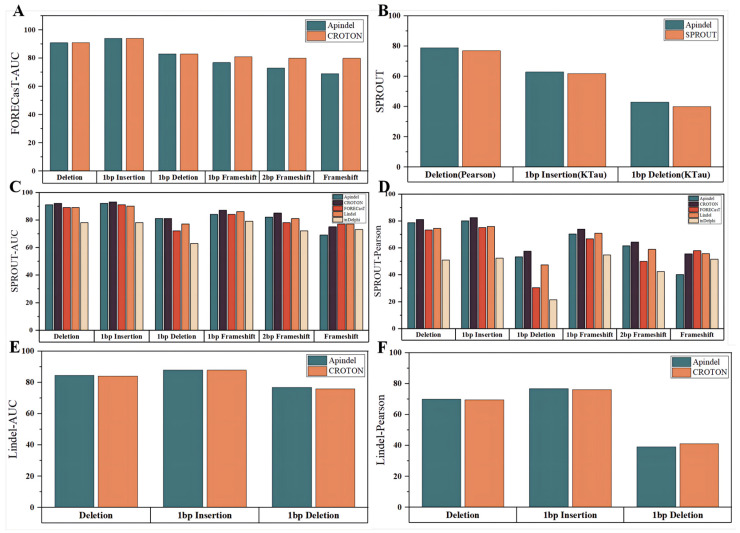
Performance comparison. The horizontal coordinates indicated the prediction tasks and the vertical coordinates indicated the prediction performance of the models on the corresponding datasets, characterized by the corresponding evaluation metrics. Different colors indicated different models. (**A**) Shows the AUC values of Apindel and CROTON on the FORECasT test set. (**B**) Indicates the comparison of the prediction performance of Apindel and SPROUT on the SPROUT test set. (**C**,**D**) Denotes the AUC values and Pearson correlation coefficients of Apindel, inDelphi, CROTON, FORECasT, and Lindel on the SPROUT test set. (**E**,**F**) Represents the comparison of Apindel and CROTON on the Lindel test set.

In the next step, Apindel was compared with other models on the CROTON test set (described in detail in the “Data sources” section). First of all, since CROTON was built based on the FORECasT dataset, we considered a comparison of the predicted edit outcomes, with the CROTON model on the FORECasT data. Apindel achieved AUCs greater than 90%, for both the deletion frequency and 1 bp insertion-frequency tasks, and AUCs of 91%,94%, and 83% for the deletion frequency,1 bp Insertion frequency, and 1 bp deletion frequency, respectively, which were the same as those of CROTON, and slightly inferior to the other models, in terms of frameshift frequency prediction (Figure 4A).

Next, in order to test the accuracy and generalization ability of Apindel in predicting DNA repair outcomes, we applied Apindel to the SPROUT dataset and the Lindel dataset, which was compared with existing machine-learning-based predictors of CRISPR/Cas9 editing results: SPROUT, inDelphi, CROTON, FORECasT, and Lindel. Given that the inDelphi, FORECasT, and Lindel models predicted more detailed repair labels, while the CROTON model predicted only six repair outcomes, including deletion frequency, we would compare model performance from the following two aspects.

On the one hand, the repair outcomes predicted by Apindel, inDelphi, FORECasT, and Lindel were organized into six repair tasks, for comparison (Figure 4C,D). Apindel outperformed other models, for most of the prediction tasks (inferior to inDelphi, FORECasT, and Lindel, for frameshift frequency). Especially in deletion frequency and 1 bp insertion frequency, Apindel achieved AUC values greater than 90% and a Pearson correlation of 80% for 1 bp insertion frequency, indicating that Apindel achieved better prediction ability.

On the other hand, for CROTON, we could, intuitively, see that on the SPROUT dataset, the prediction performance of Apindel was slightly inferior to CROTON. However, on the Lindel test set, Apindel was better than CROTON (Figure 4E,F), indicating that the prediction performance of Apindel was comparable to CROTON. It was worth noting that Apindel could predict 557 repair labels, while CROTON could predict only 6 repair labels, including deletion frequency. Apindel was more practical because it could predict the repair outcomes in a more detailed and comprehensive way. For SPROUT, the performance of Apindel was similar to or even better than SPROUT, for the three metrics (Figure 4B) thatwere trained on the SPROUT dataset. In conclusion, Apindel ran robustly on different experimental datasets, with high accuracy and generalization ability.

### 3.4. Apindel Reveals Important Sequence Loci Associated with Repair Outcomes

**Figure 5:**
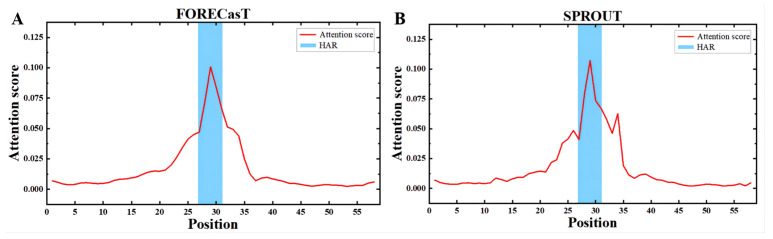
High-attention regions (HAR) [26], indicating important sequence loci for predicting repair outcomes. (**A**,**B**) The distribution of attention scores for all input sequences in the test data of FORECasT and SPROUT, showing the average importance of the different loci calculated on these sequences, for predicting repair outcomes.

An important advantage of Apindel over other deep-learning-based frameworks was that Apindel further incorporated an attention mechanism, thus allowing one to capture important sequence loci affecting predicted DNA repair, by detecting the attention vectors of samples. Here, we examined the distribution of the attention scores, by averaging the attention vector $\alpha ={\left(\alpha_{1},\cdots ,\alpha_{59}\right)}^{T}$ over all samples in the test dataset. In the FORECasT test set (Figure 5A), the High-attention regions (HAR) [26] appeared near the cut site (i.e., the 25th Kmer to the 35st Kmer), and a similar finding was shown in the SPROUT test set (Figure 5B). The above observations suggested that contextual sequences, around the 10 bp window on either side of the cleavage sites, were critical for predicting repair outcomes, and sequences farther away, relative to the cleavage sites (such as the 10th site near the $5^{\prime }$ end), had a weaker effect on repair outcomes, which was consistent with previous findings [14]. Therefore, our study confirmed that nucleotides at different locations had different degrees of effect on CRISPR/Cas9 editing outcomes, and the nucleotides near the cleavage sites had the greatest influence.

## 4. Discussion

As a simple and programmable nuclease-based genome editing tool, the CRISPR/Cas9 system has greatly improved the ability to perform precise editing in the human genome [27]. In recent years, the rapid development of CRISPR-based technology has expanded its application scope, and CRISPR technology has been applied to human diseases, cancer, plant biology [28,29,30], etc. It can be seen that the CRISPR/Cas9 system has great application prospects. Experiments demonstrated that the cellular-repair outcomes generated by CRISPR/Cas9 were determined by local-sequence features. An attention-based deep learning model was proposed, to accurately predict DNA-repair outcomes. Compared with previous models, Apindel considered more comprehensive repair labels. It can predict repair outcomes in more detail, contains a higher amount of information, and was more practical. By fully taking advantage of the superior predictive capacity of deep learning models [31] and the interpretability of the Attention mechanism [32], Apindel can, accurately, predict DNA mutational outcomes and capture the important sequence sites that affect the repair outcomes.

According to previous research [14], we knew that bases at different positions had different effects on repair outcomes. Therefore, we considered adding Positional Encoding, to represent the location information in the process of sequences encoding. In the “Model Selection” section, it can be seen that Positional Encoding had a great influence on the repair outcomes, and the Apindel model with Positional Encoding had better performance than the Apindel_NoPE model, in predicting repair outcomes, indicating the necessity of including Positional Encoding in the model.

In order to study the impact of location information on the prediction model more intuitively, we added an attention mechanism to the model. By averaging the attention vectors, we got a set of weights. Through weighting the output feature matrix, the prediction performance of Apindel was improved. By observation, we found that bases near the cleavage sites were the most important for the prediction of template-free CRISPR/Cas9 editing outcomes. It was consistent with the previous conclusion [14] and would suggest researchers should pay more attention to the impact of sequences near the cleavage sites on the repair outcomes.

Frameshift mutation refers to the deletion or addition of non-three multiples of bases in the normal DNA molecule, which causes a series of coding errors to occur after this position. Since frameshift mutation can lead to some serious consequences, we introduced the prediction task of frameshift-mutation frequency, when testing the model performance. However, to our surprise, the method based on deep learning is not doing well in predicting frameshift (e.g., Apindel and CROTON), which will become the focus of our later research work.

## 5. Conclusions

In this paper, a novel DNA-repair-outcomes prediction model, Apindel was constructed. With introduction of the GloVe model and Positional Encoding, Apindel attempted new feature representation methods, to embed sequence information into the deep learning model; the BiLSTM model was used to extract contextual sequence features, and the Attention mechanism was calculated to assign weights to the output matrix, to characterize the different influence of different loci nucleotides on the repair outcomes. The superior performance of Apindel was, further, confirmed by comparison with existing models. Our model used deep learning to comprehend the automatic learning of sequence features between DNA and corresponding repair outcomes, avoiding the unknown influence of the manual-feature-construction process on the model-prediction outcomes. Thus, it is able to predict more comprehensive and detailed repair labels, with higher accuracy and better utility. It is a new attempt of deep learning, in the direction of DNA-repair-outcome prediction.

## Figures and Tables

**Table 1:** Comparison of designs and results of CRISPR/Cas9 editing outcome prediction.

Model	Cell Line(s)	Indels Predicted ^1^	Methods ^2^
Apindel	K562	536 classes of Deletions, 21 classes of Insertions.	GloVe + Positional Encoding BiLSTM + Attention
CROTON	K562	Deletion frequency,1 bp Insertion/Deletion, 1/2 bp Frameshift frequency, Frameshift frequency.	CNN + NAS
Lindel	HEK293T	536 classes of Deletions, 21 classes of Insertions.	Logistic Regression
SPROUT	T cell	9 types statistics of the repair outcomes such as average insertion length.	Gradient Boosting Decision Tree
FORECasT	K562, RPE1, iPSC, CHOHAP1, mESCs	~420 classes of Deletions, 20 classes of Insertions.	Multi-Class Logistic Regression
InDelphi	HEK293, K562, HCT116, mESCs, U2OS	~90 classes of MH Deletion, 59 classes of Non-MH Deletion, 4 classes of 1 bp Insertion.	Deep neural network k-Nearest Neighbor

^1^ Indels predicted indicates the repair labels that can be predicted by the model. ^2^ Methods represents the main algorithms used by the model.

**Table 2:** Initial selection of the model-network framework.

Model Name	Model Description
Apindel	Final model, including all units we mentioned
Apindel_NoPE	Remove Positional Encoding from the model base
Apindel_Noattention	Remove the attention layer from the model base

**Table 3:** Performance evaluation of pre-selected models, on the Lindel dataset.

Model	Apindel	Apindel_NoPE	Apindel_Noattention
MSE	0.000164	0.000189	0.000172

## Data Availability

Datasets used during this study are included in the published article “FORECasT” (DOI: https://doi.org/10.6084/m9.figshare.7312067), “SPROUT” (DOI: https://doi.org/10.1038/s41587-019-0203-2) and “Lindel” (doi: https://doi.org/10.1093/nar/gkz487).All datasets used for this study can be found at GitHub: https://github.com/MoonLBH/Apindel.

## Supplementary Materials

The following supporting information can be downloaded at: https://www.mdpi.com/article/10.3390/cells11111847/s1, Supplementary Note; Table S1: Structure of the Apindel prediction model; Table S2: Performance comparison of pre-selected models; Table S3: Performance comparison of different models.
