# Supplementary material for: Predicting CRISPR/Cas9 Repair Outcomes by Attention-Based Deep Learning Framework
Source: Cells. 2022 Jun 5;11(11):1847. doi: 10.3390/cells11111847 (PMC9180579; doi:10.3390/cells11111847)
Supplement: Supplementary file 1 [file cells-11-01847-s001.zip › cells-1693515-supplementary.pdf]

SUPPLEMENTARY MATERIAL

Table S1. Structure of the Apindel prediction model.

Layer (type)	Description	Output Shape
embedding (Embedding)	Input dim=944, output dim=150,<br>input length=59	(59, 150)
BiLSTM (Bidirectional)	Units=50	(59, 100)
activation= "ReLU", L1 regularization(1e-04)		
Att_out1 (Dense)	Units=100	(59, 100)
activation= "tanh", L1 regularization(1e-04)		
Att_out2 (Dense)	Units=1	(59, 1)
activation= "Softmax", L1 regularization(1e-04)		
processing (processing)	tf.multiply(Att_out2.output,BiLSTM.output)	(59, 100)
flatten (Flatten)	-	(1,5900)
dense_1 (Dense)	Units=1024	(1,1024)
activation= "ReLU", L1 regularization(1e-04)		
dense_2 (Dense)	Units=557	(1,557)
activation= "Softmax", L1 regularization(1e-04)		

Table S2. Performance comparison of pre-selected models.

On Lindel data.

**Model**	Apindel	Apindel_NoPE	Apindel_Noattention
**MSE**	0.000164	0.000189	0.000172

On FORECasT data.

**Prediction tasks**	**Pre-selected models**	**AUC**	**Pearson**
Deletion frequency	Apindel	**0.91**	**0.53**
	Apindel_NoPE	0.88	0.46
	Apindel_Noattention	0.88	0.51
1 bp Insertion frequency	Apindel	**0.94**	**0.86**
	Apindel_NoPE	0.89	0.78
	Apindel_Noattention	0.91	0.83
1 bp Deletion frequency	Apindel	**0.83**	**0.70**
	Apindel_NoPE	0.80	0.58
	Apindel_Noattention	0.78	0.59
1 bp Frameshift frequency	Apindel	**0.77**	**0.43**
	Apindel_NoPE	0.75	0.37
	Apindel_Noattention	0.74	0.38
2 bp Frameshift frequency	Apindel	**0.73**	**0.46**
	Apindel_NoPE	0.72	0.43
	Apindel_Noattention	0.70	0.39
Frameshift frequency	Apindel	**0.69**	**0.26**
	Apindel_NoPE	0.66	0.16
	Apindel_Noattention	0.66	0.22

Table S3. Performance comparison of different models.

On FORECasT data.

Prediction tasks	Model	AUC
Deletion frequency	Apindel	**0.91**
	CROTON	**0.91**
1 bp Insertion frequency	Apindel	**0.94**
	CROTON	**0.94**
1 bp Deletion frequency	Apindel	**0.83**
	CROTON	**0.83**
1 bp Frameshift frequency	Apindel	0.77
	CROTON	**0.81**
2 bp Frameshift frequency	Apindel	0.73
	CROTON	**0.80**
Frameshift frequency	Apindel	0.69
	CROTON	**0.80**

On Lindel data.

Prediction tasks	Pre-selected models	AUC	Pearson
Deletion frequency	Apindel	**0.846**	**0.7**
	CROTON	0.842	0.697
1 bp Insertion frequency	Apindel	**0.881**	**0.769**
	CROTON	0.879	0.762
1 bp Deletion frequency	Apindel	**0.769**	0.392
	CROTON	0.76	**0.412**

On SPROUT data

Prediction tasks	Pre-selected models	AUC	Pearson	KTau
Deletion frequency	Apindel	0.91	0.787	-
	CROTON	**0.92**	**0.811**	-
	FORECasT	0.89	0.732	-
	Lindel	0.89	0.745	-
	inDelphi	.078	0.51	-
	SPROUT	-	0.77	-
1 bp Insertion frequency	Apindel	0.92	0.80	**0.63**
	CROTON	**0.93**	**0.824**	-
	FORECasT	0.91	0.751	-
	Lindel	0.90	0.758	-
	inDelphi	0.78	0.524	-
	SPROUT	-	-	0.62
1 bp Deletion frequency	Apindel	**0.81**	0.533	**0.43**
	CROTON	**0.81**	**0.575**	-
	FORECasT	0.72	0.304	-
	Lindel	0.77	0.473	-
	inDelphi	0.63	0.214	-
	SPROUT	-	-	0.40
1 bp Frameshift frequency	Apindel	0.84	0.703	-
	CROTON	**0.87**	**0.738**	-
	FORECasT	0.84	0.667	-
	Lindel	0.86	0.708	-
	inDelphi	0.79	0.547	-
2 bp Frameshift frequency	Apindel	0.82	0.614	-
	CROTON	**0.85**	**0.643**	-
	FORECasT	0.78	0.50	-
	Lindel	0.81	0.589	-
	inDelphi	0.72	0.424	-
Frameshift frequency	Apindel	0.69	0.402	-
	CROTON	0.75	0.556	-
	FORECasT	**0.77**	**0.579**	-
	Lindel	**0.77**	0.557	-
	inDelphi	0.73	0.515	-

SUPPLEMENTARY NOTE

- **Data preprocessing**

In the data pre-processing stage, we consider truncating the genomic inputs to 60 bp for several reasons: first, among published papers, such as CROTON and the Lindel model, 60 bp genomic sequenceswere considered as inputs to the model. Second, based on the previous research findings that "repairoutcomes can be predicted by the local context of the sequences", we chose to truncate the 30 bp sequences above and below the cut sites.

When we truncate the sequences with the cut sites as the center, the lengths of the truncated sequences are often less than 60 bp. Specifically, for reverse sequences, there will be three bases missingupstream, so we need to pad three bases. For forward sequences, there will be four bases missing downstream, so we need to pad four bases. For padded-base types, CROTON chose to pad the FORECasT sequences with the pseudo-letter 'N'. In fact, since sequences that are further away fromthe cleavage sites have little effect on the repair outcomes, padding different base types, also, has little effect on the outcomes (see Table 1). In this paper, we choose to add "ATG" before the reverse sequences and "ATGC" after the forward sequences.

Table 1. AUC values of models with different padding sequences on the FORECasT dataset.

padded sequences	padded sequences	Deletion	1 bp Insertion	1 bp Deletion	1 bp Frameshift	2 bp Frameshift	Frameshift
ATG	ATGC	0.911	0.936	0.83	0.769	0.729	0.686
TCC	CGTA	0.909	0.935	0.829	0.773	0.73	0.689

- **Description of AUC**

In this paper, AUC refers to the area under the ROC curve, ranging from 0 to 1. The larger the AUC value is, the higher the accuracy of the model. The ROC curve is drawn by the following process: first, make the test data of each prediction task equal to 1 (test data larger than or equal to median) or equal to 0 (test data lower than median), and convert the original test data to "0-1" data, by the above process. Next, the samples are sorted, according to the predicted probability output value of all samples, and, then, the probability output value is used as the threshold for classification from the top to the bottom. The false positive rate (FPR) and true positive rate (TPR) are calculated by the following formula, and the horizontal coordinate is set as the false positive rate (FPR), the vertical coordinate is set as the true positive rate (TPR), and the ROC curve is drawn.

$$TPR = \frac{TP}{TP + FN} \tag{1}$$

$$FPR = \frac{FP}{FP + TN} \tag{2}$$
